# Synthesis of vancomycin functionalized fluorescent gold nanoparticles and selective sensing of mercury (II)

**DOI:** 10.3389/fchem.2023.1238631

**Published:** 2023-08-01

**Authors:** Atul Kumar Tiwari, Hari Prakash Yadav, Munesh Kumar Gupta, Roger J. Narayan, Prem C. Pandey

**Affiliations:** ^1^ Department of Chemistry, Indian Institute of Technology, Banaras Hindu University, Varanasi, India; ^2^ Department of Microbiology, Institute of Medical Sciences, Banaras Hindu University, Varanasi, India; ^3^ Joint Department of Biomedical Engineering, University of North Carolina, Chapel Hill, NC, United States

**Keywords:** functional gold nanoparticles, polyethyleneimines, vancomycin-loaded gold nanoparticles, fluorescent gold nanoparticles, fluorometric sensing

## Abstract

Mercury ions (Hg^2+^) are widely found in the environment; it is considered a major pollutant. Therefore, the rapid and reliable detection of Hg^2+^ is of great technical interest. In this study, a highly fluorescent, sensitive, and selective fluorometric assay for detecting Hg^2+^ ions was developed using vancomycin functionalized and polyethyleneimine stabilized gold nanoparticles (PEI-f-AuNPs@Van). The as-made gold nanoparticles were highly fluorescent, with excitation and emission maxima occurring at 320 and 418 nm, respectively. The size of nanoparticles was ~7 nm; a zeta potential of ~38.8 mV was determined. The XRD analysis confirmed that the nanoparticles possessed crystalline structure with face centerd cubic symmetry. Using the PEI-f-AuNP@Van probe, the detection limit of Hg^2+^ ion was achieved up to 0.988 nM (within a linear range) by calculating the KSV. However, the detection limit in a natural environmental sample was shown to be 12.5 nM. Furthermore, the selectivity tests confirmed that the designed probe was highly selective to mercury (II) cations among tested other divalent cations. Owing to its sensitivity and selectivity, this approach for Hg^2+^ ions detection can be utilized for the analysis of real water samples.

## Graphical Abstract

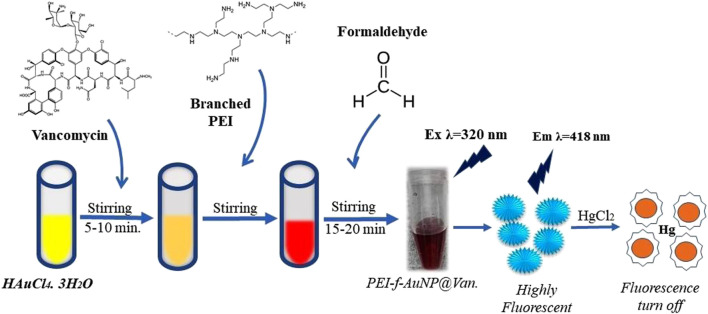

## 1 Introduction

Mercury ion (Hg^2+^) is an environmental metal contaminant; it is a nonessential and is toxic to lower organisms and humans. In case of human exposure, it can accumulate in body by the kidneys and resulting symptoms could include vomiting, diarrhea, hypovolemic shock, kidney failure, and potential death (Cowan, 1997; Park and Zheng, 2012; Fang et al., 2014; Gibb and O’Leary, 2014). It should also be noted that inorganic mercury ions and organic mercury ions (e.g., methyl mercuric (MeHg^+^), phenylmercuric (PhHg^+^), and ethyl mercuric (EtHg^+^) ions) can cause neurologic problems (e.g., ataxia, visual field constriction, hearing impairment, and blindness) (Hong et al., 2012; Dorea et al., 2013; Geier et al., 2014). As such, rapid and reliable mercury (II) ion detection is of technical interest. A variety of approaches such as direct mercury analysis (DMA) (Windmoller et al., 2017), Ion chromatography (IC) (Liu, 2010), as well as high-performance liquid chromatography (HPLC) (Yin et al., 2010; Cheng et al., 2014) have been demonstrated for mercury ion detection. It should be noted that these approaches involve complicated protocols such as sample pre-treatment steps, skilled technicians, and sophisticated tools. As such, the implementation of a low-cost and facile analytical approach for selective mercury ion detection is a major focus area in the field of analytical chemistry. Several types of nanoparticle assays that offer straightforward detection of Hg^2+^ ions have been evaluated; these approaches involve gold nanorod structures (Au-NR) (Placido et al., 2013), carbon nanoparticles (CNP) (Guo et al., 2013), silver nanoparticles (Ag-NPs) (Li et al., 2015), silver nano prism structures (Ag-NPR) (Chen et al., 2016), and gold nanoparticles (Au-NPs) (Chen et al., 2014; Sener et al., 2014; Chen et al., 2015). Due to inter-particle distance-dependent optical characteristics and high extinction coefficients, these colorimetric methods offer the capability of analyzing Hg^2+^ visually or via UV-Vis instrumentation.

Among various types of nanomaterials, gold nanoparticles (Au-NPs) have been recognized for their utility in various sensing and imaging applications. One of the desirable optical properties of Au-NPs is their localized surface plasmon resonance (LSPR) characteristics. Lorenz-Mie scattering was used to explain the SPR characteristics of spherical Au-NPs (Mulvaney, 1996; Sardar et al., 2009) and confirmed that fluorometric behavior of Au-NPs is depends on their particle shape, particle size, nanoscale geometry, refractive index of the medium, inter-particle distance, and aggregation state in a given solution. Any variation in these factors affects the plasmon-resonance frequency characteristics (Daniel and Astruc, 2004; Lin et al., 2011). These parameters may explain why Au-NPs having a size 5–20 nm appear deep red in color, while aggregates of small particles or larger particles appear deep blue to purple in color.

Considering the unique characteristics of Au-NPs, fluorometric sensing methods have attracted the research community due to the benefits, which include rapid analysis, cost-effective, and straightforward operation (Ma et al., 2017; Kim et al., 2018; Sedgwick et al., 2018; Vellaisamy et al., 2018; Wang et al., 2018; Wu et al., 2018). Further, Au-NPs have been widely used as a quenching material owing to their unique properties such as a large surface area to volume ratio, straightforward surface functionalization, and LSPR absorption characteristics in the visible light region (Furletov et al., 2017). The functionalization approach utilized with Au-NP materials involves surface modification with either ligands or receptor molecules using gold-sulfur (Au-S) and gold-nitrogen (Au-N) bonds. The use of a surface functionalization approach to alter the fluorescence activity of Au-NPs has been considered in this study. Under specific conditions (e.g., at high temperatures), PEI- mediated formation of gold nanoparticles over a relatively longer period of time has been previously discussed; however, we have for the first time described the rapid and controlled synthesis of gold nanoparticles under ambient conditions, in which there is an active role for PEI and formaldehyde/cyclohexanone (Pandey and Pandey, 2016; Pandey et al., 2017a; Pandey et al., 2017b). An advantage of the benign approach described in this study is that it is compatible with gene delivery mediators, templates, and stabilizers for modifying metal nanoparticles (Kumar et al., 2015). For example,. Amine groups may provide sufficient active sites for functionalization; these features of PEI can facilitate changes to the selectivity of various ions and biomolecules to deliver drugs and genes for breast cancer therapy and to deliver antimicrobial agents (Li et al., 2015; Kumar et al., 2015; Tiwari et al., 2020; Tiwari et al., 2022). Furthermore, Polyethyleneimine has been utilized for synthesizing noble metal nanoparticles (Tiwari et al., 2020; Tiwari et al., 2022; Pandey et al., 2023) and functionalization with curcumin (Pandey et al., 2023). These studies directed us to consider vancomycin, a potent agent used in the treatment of severe bacterial infections in hospitalized patients, which is a green-fluorescent analog**.** We described novel findings related to the formation of highly fluorescent gold nanoparticles via active participation of PEI, vancomycin, and formaldehyde; the fluorescent activity of the gold nanoparticles was entirely different from those of the respective precursors. Dramatically, as made fluorescent gold nanoparticles displayed selective variation of their fluorescent activity as a function of mercuric ions; findings on this topic are reported herein.

## 2 Materials and methods

All of the materials and reagents were analytical quality. Vancomycin (purity ≥85%, CAS no. 1404-93-9) Polyethyleneimine (50% w/v in H_2_O; CAS no. 9002-98-6), tetra-chloroauric acid trihydrate (HAuCl_4_.3H_2_O; purity 99.9%, CAS no. 16961-25-4), and formaldehyde (≥36.0% in H_2_O) were purchased from Sigma Aldrich (Mumbai, India). Mercuric chloride (purity; ≥99.5%), potassium chloride (purity; 99%), sodium chloride (99%), magnesium chloride, and ammonium chloride were purchased from Merck (Bangalore, India). Other required glassware and plastic wares were purchased from Tarson (Mumbai, India). All of the experiments were performed using ultra-purified HPLC-grade water.

### 2.1 Synthesis of vancomycin-loaded polyethyleneimine functionalized gold nanoparticles

The PEI functionalized gold nanoparticles were synthesized per the procedure that was previously reported with slight modification (Pandey et al., 2023). Briefly, 400 μL (10 mM) of hydrogen tetra-chloroauric acid trihydrate (HAuCl_4_.3H_2_O) was placed in a 2 mL glass vial; this step was followed by the addition of an aqueous solution of vancomycin (40 μL, 2 mg/mL stock). The reaction mixture was then stirred on a magnetic stirrer for 5–10 min, followed by the addition of an aqueous solution of Polyethyleneimine (30 μL of 4 mg/mL stock solution). The reaction mixture continued to be mixed on the stirrer, followed by the addition of 30 μL of formaldehyde; this mixture continued to be stirred for the next 30–60 min to yield dark red-pink color PEI-f-AuNP@Van.

### 2.2 Physical characterization of synthesized nanoparticles (PEI-f-AuNP@Van)

Several characterization methods were used to understand the synthesized fluorescent gold nanoparticles (Au-NPs). UV-VIS spectroscopy measurements were obtained using a Hitachi U-2900 spectrophotometer (Tokyo, Japan). Transmission electron microscopy (TEM) measurements were acquired using a FEI Tecnai G2 20 S Twin instrument (Hillsboro, Oregon, United States). The average length of gold nanoparticles was measured using ImageJ software (National Institutes of Health, Bethesda, MD, United States); a statistical graph was plotted on Origin 8.5 software (Northampton, MA, United States). The fluorescence emission (FL emission) spectra were obtained with a Hitachi F7000 fluorescence spectrophotometer (Tokyo, Japan). An X-ray photoelectron spectroscopy (XPS) instrument (Thermo Fisher Scientific, Waltham, MA, United States) with a K-alpha source was used to determine the binding energy and chemical composition of the samples. XRD spectra obtained using a Rigaku Mini-Flex 600 instrument (Tokyo, Japan). Time-resolved fluorescence spectra were acquired using a WiTec alpha 300 RA instrument (Ulm, Germany); a Malvern Nano Zeta Sizer (Malvern, United Kingdom) was used to obtain dynamic light scattering (DLS) zeta potential data.

### 2.3 Sensing of mercury ion (Hg^2+^)

All of the experiments were carried out in ultra-purified HPLC grade water. A 10 μL of synthesized gold nanoparticles were added to an aqueous solution containing various HgCl_2_ concentrations (ranging from 2 to 64 μM), which were placed in a 1 cm quartz cuvette; fluorescence spectra were recorded from these samples. All of the fluorescence spectra were obtained with a 5/10 slit width using an excitation of 320 nm. All of the measurements were repeated three times to assess if the results were accurate and the method was consistent.

### 2.4 Detection of mercury ion (Hg^2+^) in an actual environmental sample

A water sample was obtained from a pond at the institute campus (Indian Institute of Technology, BHU) Varanasi, India; this samples used immediately as an environmental sample without filtration. Various calculated spiked concentrations of HgCl_2_ (ranging from 0.5 to 32 μM) were added to a 1 cm quartz cuvette that contained a fixed volume of environmental sample and fluorescent Au-NPs; fluorescence spectra were recorded from these samples (Ma et al., 2019).

## 3 Results and discussion

### 3.1 Synthesis and characterization of vancomycin-loaded gold nanoparticles

**FIGURE 1:**
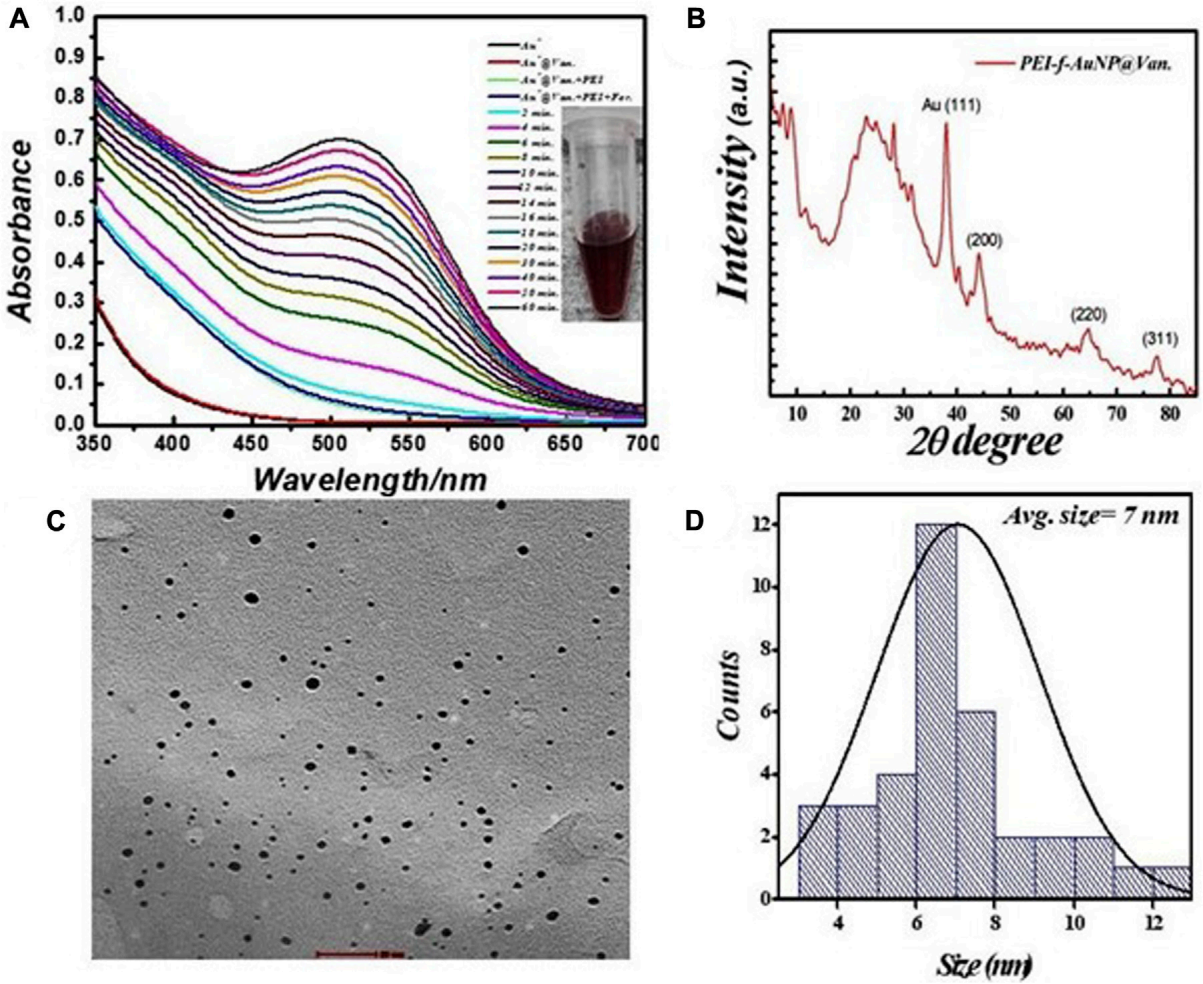
**(A)** Real time UV–visible spectra of vancomycin-loaded Polyethyleneimine functionalized Au- NPs synthesis, **(B)** corresponding X-ray differactogram, **(C)** TEM micrograph, and **(D)** size distribution plot.

The prepared PEI-f-AuNPs were ∼7 nm in size using HAuCl4.3H2O As described in previous studies, Au- NPs synthesized by reducing HAuCl_4_ with citrate and other organic reducing agents (e.g., cyclohexanone) using a 3-glycedoxypropyltrimethoxysilane-mediated reduction were 10–33 nm (Rak et al., 2014; Mitra and Pandey, 2022); Au-NPs conjugated to branched Polyethyleneimine (PEI) with a molecular weight of 750 k Da under these conditions were much smaller (5–7 nm). As such, the mean Au-NP size was dependent on both the type and quantity of reducing agents, temperature, pH, and reaction time (Lin et al., 2013; Deraedt et al., 2014). A straightforward protocol was undertaken to reduce the gold cation in order to produce vancomycin-loaded gold nanoparticles; PEI was used as a stabilizing agent and formaldehyde was used as a reducing agent. The synthesized PEI-f-AuNP@Van were characterized using UV-Vis spectrophotometry; monitoring over time indicated a strong absorbance peak at 520 nm (Figure 1A), which was attributed to the synthesis of small-sized gold nanoparticles. TEM characterization confirmed the actual size of vancomycin-loaded gold nanoparticles was ∼7 nm (Figures 1C, D).

**FIGURE 2:**
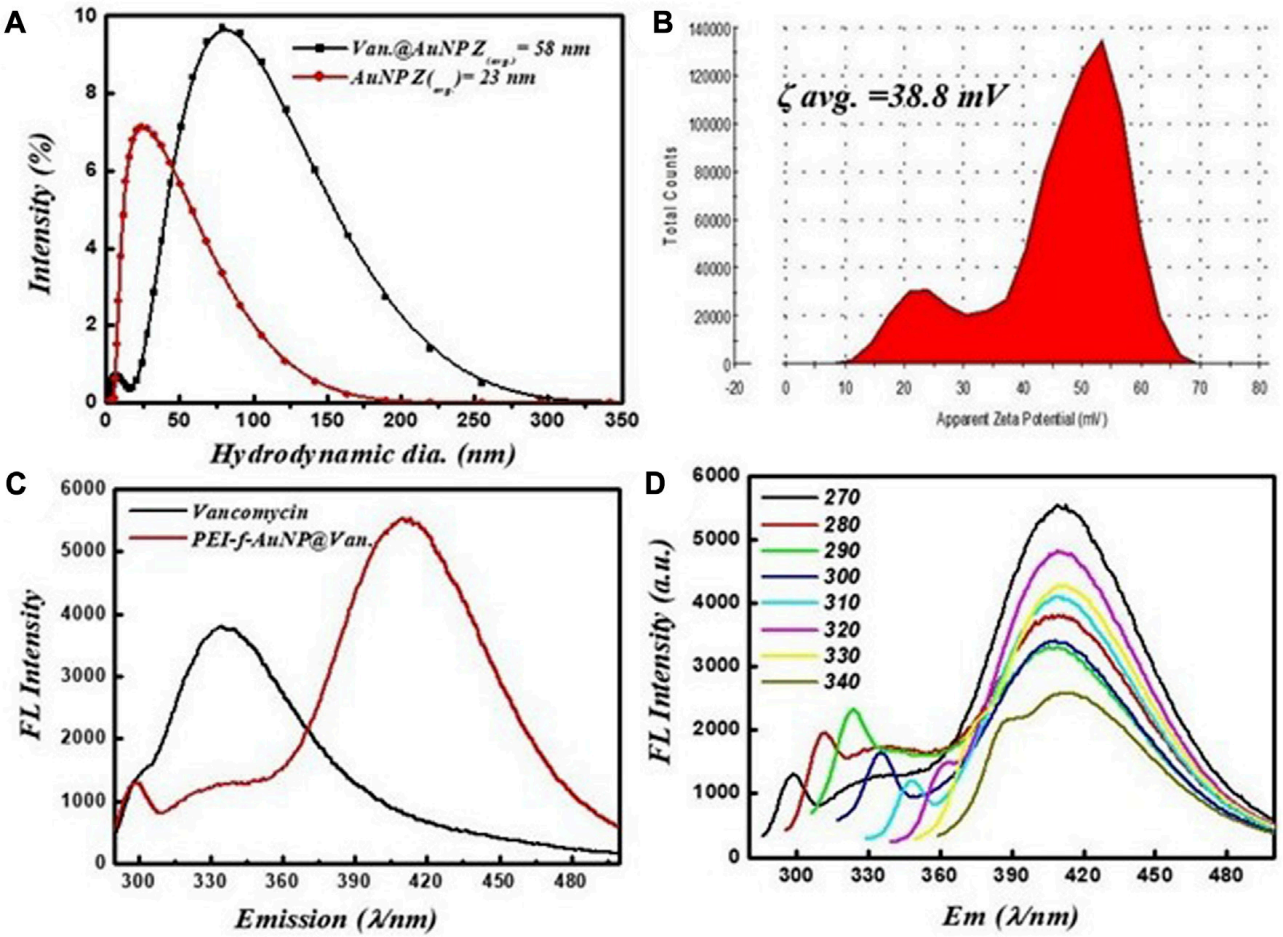
**(A)** Hydrodynamic radii of vancomycin-loaded and unloaded gold nanoparticles, **(B)** zeta potential, **(C)** fluorescent spectra of vancomycin and vancomycin-loaded gold nanoparticles excited at 270 nm, and **(D)** fluorescence screening of vancomycin-loaded gold nanoparticles at various excitation wavelengths (270–340 nm).

The crystallinity and microstructure of the synthesized PEI-f-AuNP@Van were investigated using XRD from 10° to 80° 2θ degrees. Figure 1B shows the peaks for fluorescent Au-NPs as 38.4°, 44.6°, 64.6°, and 77.7°, corresponding to hkl values (111), (200), (220), (311) lattice planes, respectively. The peak at 38.4° was more intense than the other peaks, which suggested that Au in the fluorescent Au-NPs was in the face-cantered unit cell (FCC) structure. The hydrodynamic radii of fluorescent PEI-f-AuNP@Van were determined as ∼58 nm, which is larger than that of the bare Au-NPs (∼23 nm) as shown in Figure 2A. The increase in the hydrodynamic radii was associated with the loading of vancomycin. As shown in Figure 2B, the zeta potentials were to be ∼38.8 mV, indicating the presence of a positive charge on the surface of the fluorescent Au-NPs. Because of the greater magnitude of the zeta potential value, the dispersibility of the Au-NPs in water over many months was maintained, which indicates the stability of the fluorescent Au-NPs.

### 3.2 Fluorescence properties of functionalized gold nanoparticles

The fluorescence activity of vancomycin-loaded Au-NPs was evaluated to get a better understanding of the impact of the synthesis protocols on the applications of the materials. The emission spectra of the vancomycin-loaded gold nanoparticles at a given wavelength (*λ* = 280 nm) are shown in Figure 2C. It was revealed that the excitation of vancomycin at 270 nm was associated with an intense emission at 335 nm; when loaded on the gold nanoparticles, the emission shifts to 418 nm with significant fluorescence intensity. As shown in Figure 2C, the results indicate that the fluorescence activity was a function of vancomycin and the nanoscale geometry of gold. The fluorescence emission spectra indicate the PEI-f-AuNP@Van emits blue light when it is exposed to UV light with *λ* = 270 nm. A single absorption peak was noted at 520 nm from the dispersion of Au-NPs; an emission peak appeared at 418 nm after excitation at 270 nm. The fluorescent Au-NPs exhibited excitation-dependent activity over the 270–340 nm range as indicated in Figure 2D. During the early stages of the study, the wavelength of the excitation light decreased from 280 to 300 nm; the FL emission intensity of the light progressively increased without emission wavelength shifting. As the experiment progressed over time, the emission intensity progressively decreased. Our results indicate that diverse trap surface states cause an excitation-dependent emission pattern in the as-made fluorescent Au-NPs.

### 3.3 XPS analysis

**FIGURE 3:**
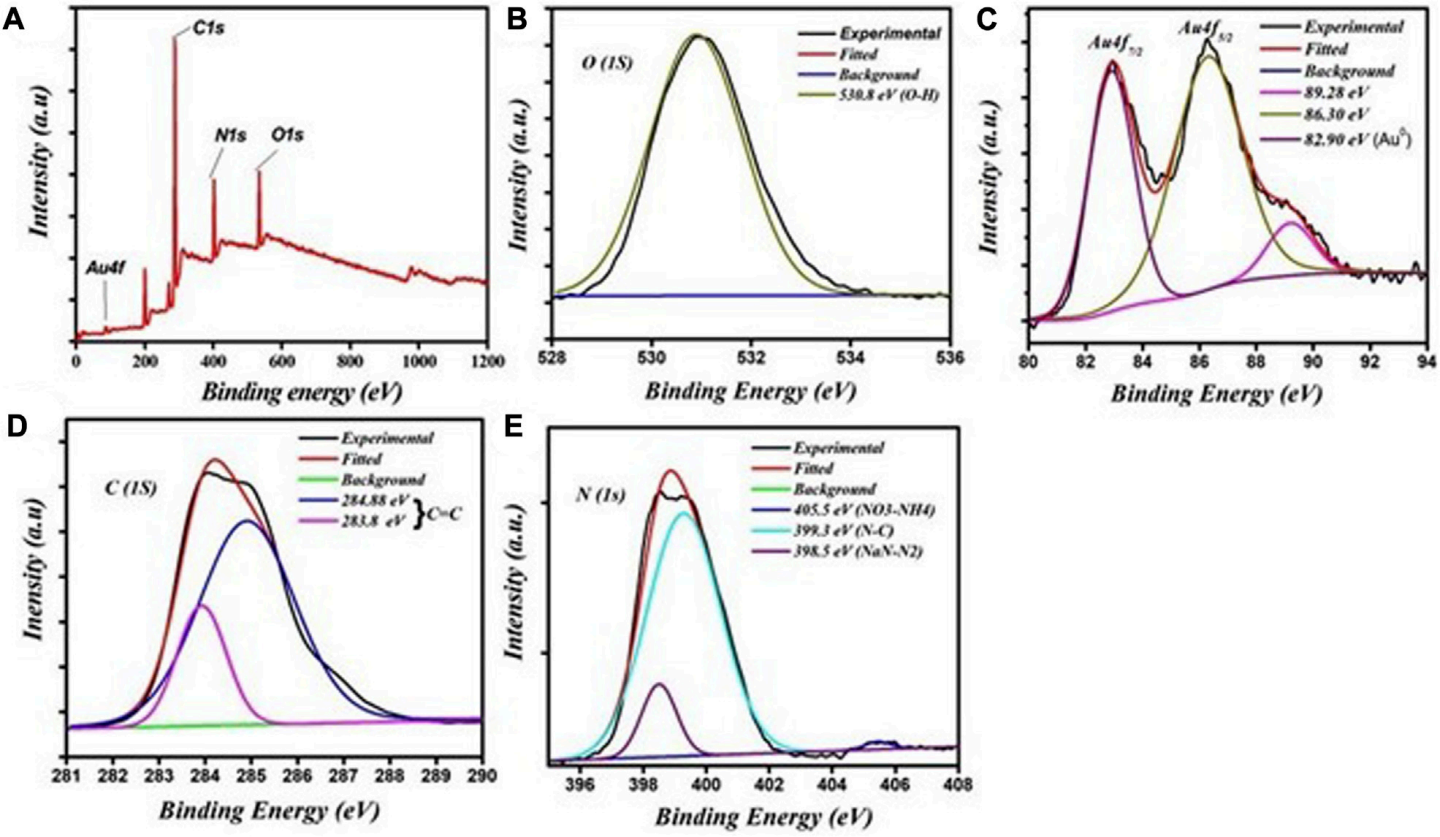
XPS spectra of the fluorescent Au-NPs: **(A)** survey scan showing the presence of various elements, **(B)** O1s spectra, **(C)** Au 4f spectra, **(D)** C1s spectra, and **(E)** N1s spectra.

XPS measurements were undertaken to examine the chemical composition and the chemical bonding in the synthesized materials. Figure 3A shows the XPS survey spectrum of the fluorescent Au-NPs, which indicates that C, N, O, and Au were present. A deconvolution procedure on these peaks was undertaken to evaluate the chemical bonding as shown in Figures 3B–E. The XPS C1s, N1s, and O1s spectra are shown in Figures 2B, D, E, respectively. Figure 3C shows the XPS Au 4f core level spectrum. The oxidation state of the gold atom in HAuCl_4_ was initially Au^3+^; after PEI and formaldehyde were added to the HAuCl_4_ solution, the gold atom oxidation state decreased from Au^3+^ to Au^0^.

### 3.4 PEI-f-AuNP@Van mediated fluorescence sensing of Hg^2+^

**FIGURE 4:**
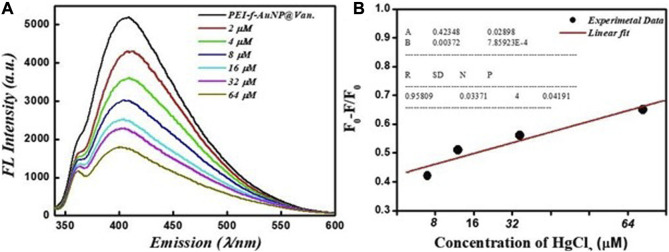
**(A)** Dependence of Fluorescence emission intensity on Hg^2+^ concentration between 2 and 64 μM, and **(B)** Stern–Volmer (S–V) plot with the addition of Hg^2+^ under similar conditions into vancomycin loaded Au-NPs. The inset to **(B)** shows the kinetic parameters.

The results shown in Figures 2C, D indicate the excellent fluorescent activity of vancomycin-loaded gold nanoparticles. We then evaluated the functionality of the as-made fluorescent gold nanoparticles on mercury (II) sensing using PEI-f-AuNP@Van as a probe (shown in Figure 4A). The results showed that the Hg^2+^ cation significantly quenched the fluorescence emission of the developed probe. After the concentration of the Hg^2+^ cation was raised from 2 to 64 μM, the fluorescence intensity of PEI-f- AuNP@Van was noted to be considerably quenched (as indicated in Figure 4A). It is again important to note the kinetic variation (if any) on the activity of PEI-f-AuNP@Van on Hg^2+^-based fluorescence quenching, which is based on the Stern–Volmer equation:

$$F0/F=1+KSV\;\left[{Hg}^{2+}\right]$$ (1)

In this equation, F is the FL intensity as a function of various concentrations of the quencher [Hg^2+^], F_0_ is the FL emission intensity at [Hg^2+^] = 0, and K_SV_ is the Stern–Volmer quenching constant. The relative kinetic variation determined using the above equation is plotted in Figure 4B. The kinetic parameters are incorporated into Figure 4B for the nanoparticles, which show the dependence of fluorescence quenching of PEI-f-AuNP@Van on Hg^2+^ sensing. The LOD was determined; it was noted that it might be as low as 0.988 nM (calculated at a DL = 3.3 × σ/S, where *σ* = slope and S = standard deviation). This finding demonstrates that PEI-f-AuNP@Van show promise for as a sensitive approach for Hg^2+^ detection.

### 3.5 Plausible mechanism for binding of Hg^2+^ to the PEI-f-AuNP@Van and physical changes after addition of Hg^2+^

**FIGURE 5:**
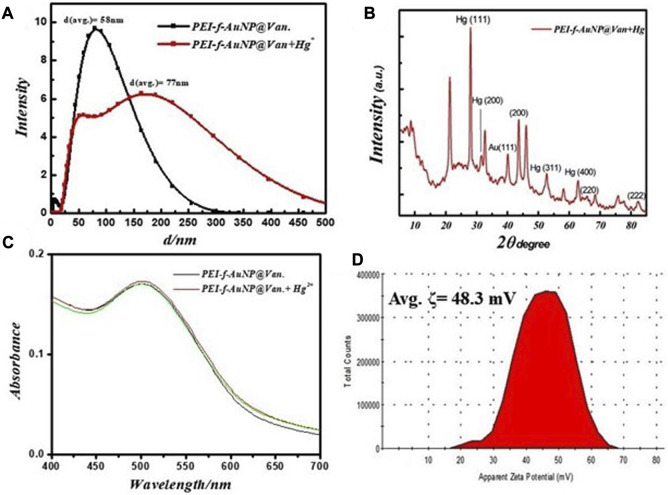
**(A)** Changes in fundamental physical properties of vancomycin loaded Au-NP after the addition of Hg^2+^ under similar conditions, hydrodynamic radii, **(B)** XRD result, **(C)** UV-Visible spectrum, and **(D)** zeta potential.

The physical characteristics of the developed probe were monitored by considering parameters such as the effect of Hg^2+^ ions on hydrodynamic radii, the effect on UV-Vis absorbance of the probe, as well as changes in crystallinity and zeta potential. The results showed that the hydrodynamic radii of synthesized gold nanoparticles were ~58 nm (as shown in Figure 2A); the radii increased to ~77 nm after a fixed concentration of mercury ions was added with decreasing count percent (Figure 5A). The increase in hydrodynamic radii indicated the binding of mercury ions to the gold nanoparticle surface. This binding could be mediated by the nitrogen atoms of PEI and vancomycin as suggested by a previous study that was conducted by Kim et al. (2018) ^.^ Further, the N 1 s feature at 406.2 eV in Hg^2+^-PEI-AuNPs indicated the binding energy of the Hg^2+^-N bonds (Tao, 2012). Another study confirmed the binding of Hg^2+^ to the tertiary nitrogen of PEI-Au-NP; however, the role of vancomycin in the binding of Hg^2+^ is not confirmed yet except for making the gold nanoparticles highly fluorescent. The XRD pattern of Hg^2+^ ions added to fluorescent gold nanoparticles was obtained (Figure 5B); this result confirms the coordinated binding of Hg^2+^ ions to the nanoparticles. Intense XRD peaks of mercury were observed at the planes of (111), (200), (311), and (400) except for gold nanoparticles (as shown in Figure 5B). The UV-Vis spectrum of gold nanoparticles was recorded to observe any change in absorbance in PEI-f-AuNP@Van after the addition of Hg^2+^ ions (as indicated in Figure 5C). However, no such phenomena were observed. Further, the zeta potential of PEI-f- AuNP@Van recorded after adding Hg^2+^ increased significantly to ~48.3 mV as shown in Figure 5D compared to bare PEI-f-AuNP@Van (Figure 2D; 38 mV). This increase in zeta potential enhanced the stability of the probe and confirmed once again the binding of Hg^2+^ to the probe.

### 3.6 Selectivity of PEI-f-AuNP@Van for Hg^2+^ ions and stability at various parameters

**FIGURE 6:**
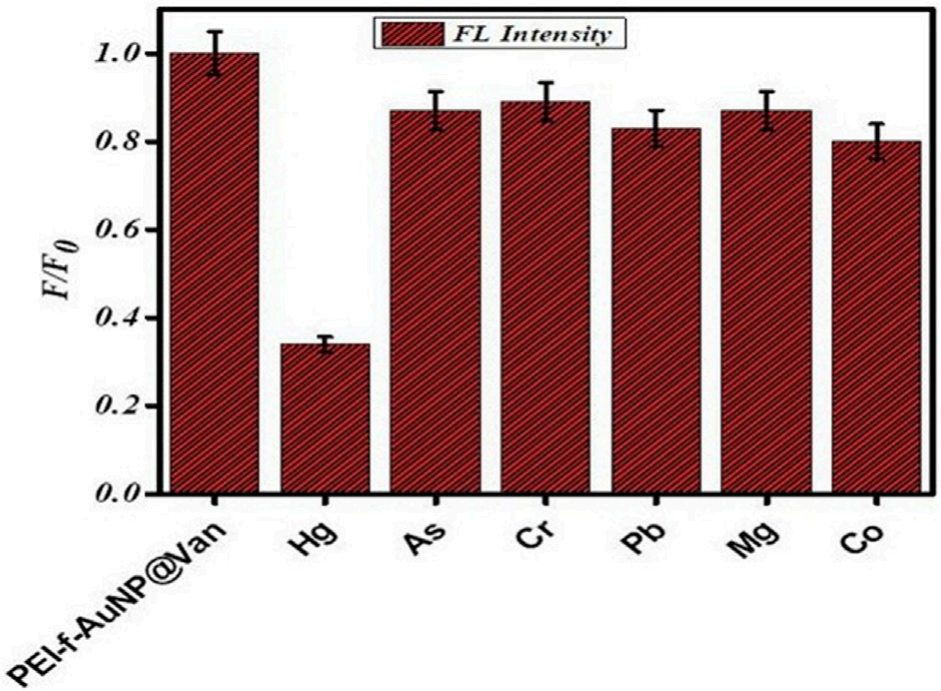
Selectivity of PEI-f-AuNP@Van against the tested heavy metal analytes.

The selectivity 0f the PEI-f-AuNP@Van assay was evaluated with 60 μM Hg^2+^ and various metal cations (100 μM Cr^3+^, Pb^2+^, As^3+^, Mg^2+^, and Co^2+^ ions). As shown in Figure 6, only Hg^2+^ showed significant fluorescence quenching behavior against the probe among the tested metal cations. The Hg^2+^ quenched the fluorescence of PEI-f-AuNP@Van around 70%; the other tested anions quenched only 10%–20%. The high quenching ratio of Hg^2+^ was associated with the aggregation of PEI-f-AuNP@Van; a low quenching ratio in the presence of other metal ions indicated the maintenance of well-dispersed forms of PEI-f-AuNP@Van. As such, the results suggest that the Hg^2+^ ion is selectively coordinated with sites on PEI-f-AuNP@Van.

**FIGURE 7:**
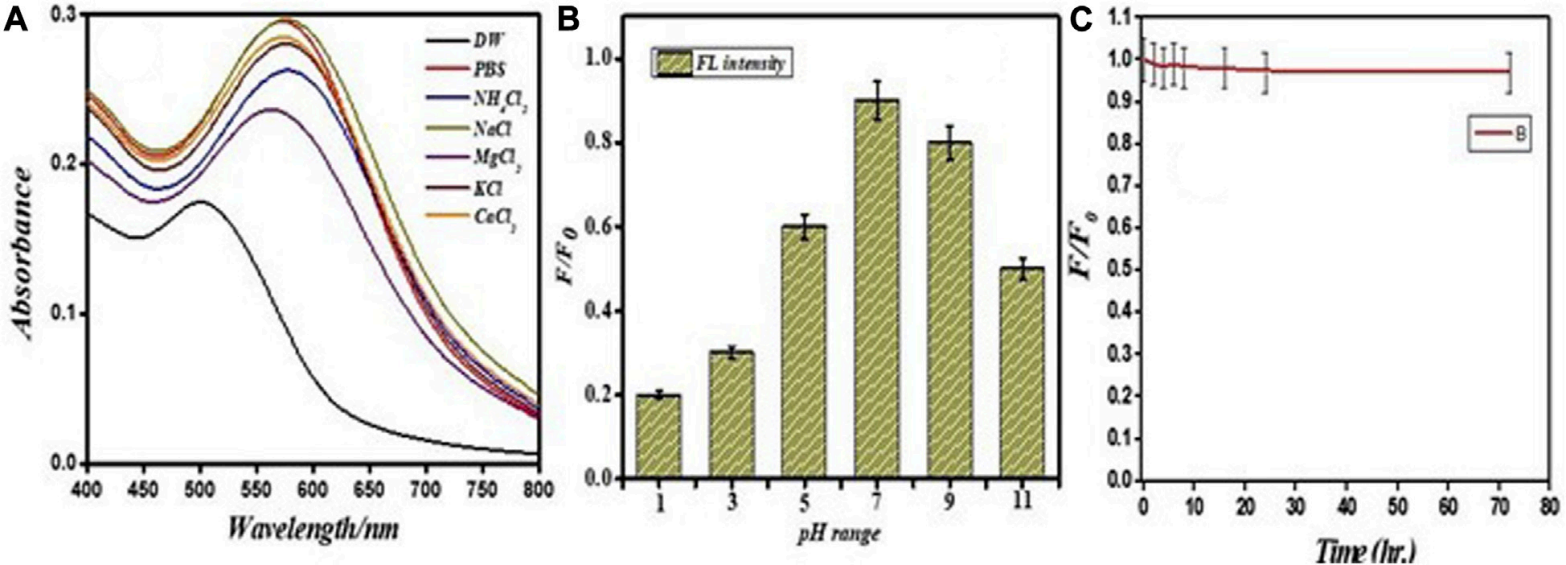
Stability of PEI-f-AuNP@Van in different salts **(A)** on variable pH **(B)** and effect of time on the fluorescence **(C)**.

The probe was assessed as a function of salt tolerance, pH, and the effect of time on the fluorescence property of the probe to optimize the sensitivity of the PEI-f-AuNP@Van probe for Hg^2+^. The fluorescence intensity of the PEI-f-AuNP@Van probe was modulated as a function of pH; this value was the highest at pH = 7.0 (Figure 7B). This optimum pH of the PEI-f-AuNP@Van probe is likely related to the conformation of PEI and the pKa of the tertiary amine (Curtis et al., 2016). At pH 7, the Hg^2+^ must be optimally coordinated with nitrogen atoms in PEI in its N-tetrahedral form, giving rise to highest sensitivity and selectivity for the probe (GaJney and Marley, 2014).

### 3.7 Real sample analysis

**FIGURE 8:**
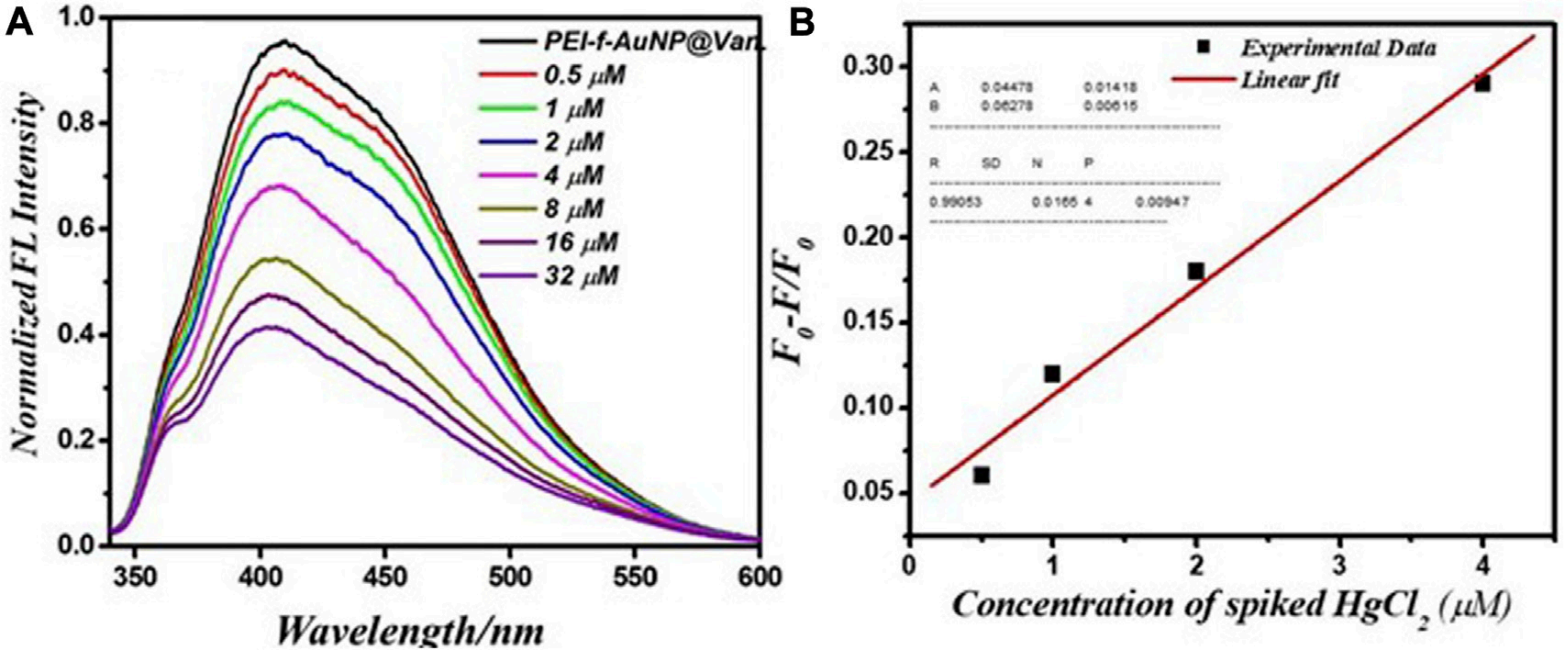
**(A)** Relationship between the fluorescence emission intensity and spiked Hg^2+^ concentration between 0.5 and 32 μM, and **(B)** Stern–Volmer (S–V) plot with addition of Hg^2+^ under similar conditions into vancomycin-loaded Au-NPs. The inset to **(B)** shows the kinetic parameters.

Pond water was used to evaluate the performance of PEI-f-Au-NP@Van for detecting Hg^2+^. The pond water samples were spiked with Hg^2+^ (ranging from 0.5 to 32 μM). The results indicate that the Hg^2+^ cation significantly quenched the fluorescence emission of the probe. After the concentration of the Hg^2+^ cation was increased from 0.5 to 32 μM, the fluorescence intensity of PEI-f-AuNP@Van was noted to be markedly quenched (Figure 8A). It is important to consider the potential role, if any, of kinetic variation on the performance of PEI-f-AuNP@Van on Hg^2+^ fluorescence quenching based on the Stern Volmer equation as provided above; the relative kinetic variation based on the Stern–Volmer equation is plotted as provided in Figure 8B. The kinetic parameters are inserted in Figure 8B for the nanoparticles, indicating the dependence of fluorescence quenching of PEI-f-AuNP@Van on Hg^2+^ sensing. The LOD was calculated; it might be as low as 12.5 nM (calculated at a DL = 3.3 *×* σ/S). This demonstrates that PEI-f-AuNP@Van is a promising material for sensitive Hg^2+^ detection. A detailed quantitative study is currently underway.

### 3.8 Fluorescence lifetime decay analysis

The fluorescence quenching of PEI-f-AuNP@Van in the presence of Hg^2+^ was evaluated using a time-resolved fluorescence decay analysis; this approach was used to determine quenching. The fluorescence decay can be evaluated using a double exponential function with the following equation:

$$D\;\left(t\right)=\sum_{i=1}^{n}ai\;\exp\left(-\frac{t}{\tau i}\right)$$ (2)

**FIGURE 9:**
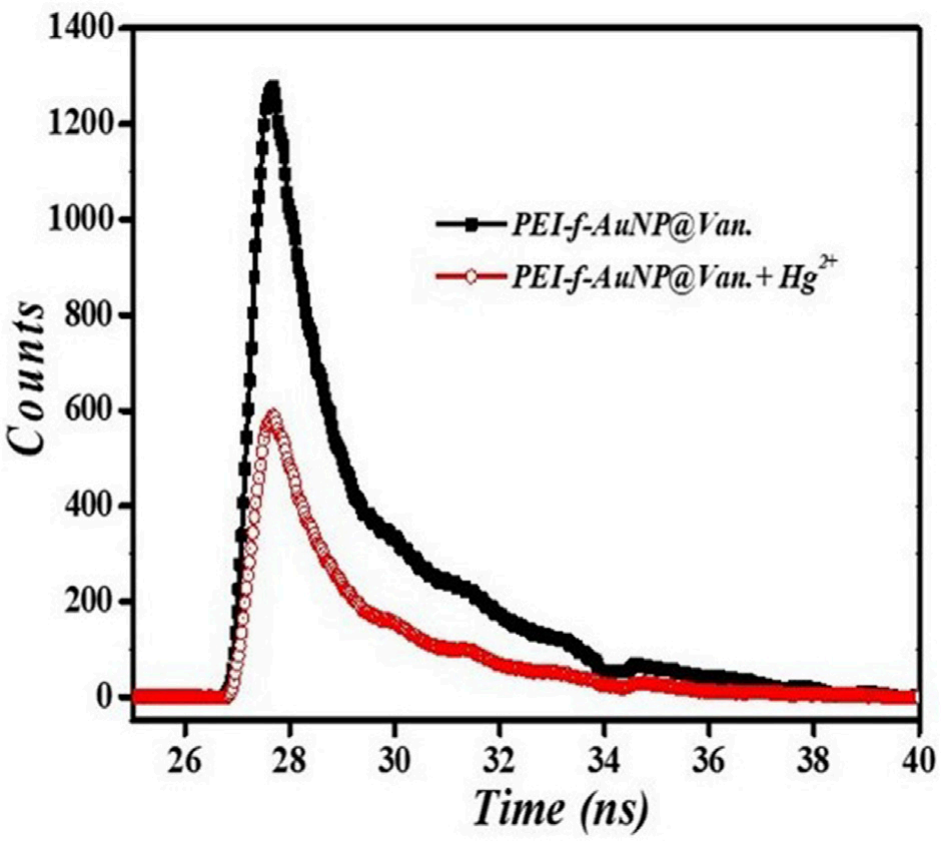
Time resolved fluorescence lifetime of PEI-f-AuNP@Van incubated with and without Hg^2+^.

In the equation, τi represents the fluorescence lifetimes of numerous fluorescent forms; D represents the fluorescence decay, and *ai* representing the associated pre-exponential factors (Raut et al., 2014). When no acceptor is present, the PEI-f-AuNP@Van shows an average lifetime of 3.25 ns (as indicated in Figure 9). The average lifetimes of 1.12 ns was significantly lower after addition of electron acceptor Hg^2+^, which indicates that PEI-f-AuNP@Van transfers electrons to Hg^2+^; it serves as an electron acceptor to lower the intensity of the fluorescence emission of PEI-f-AuNP@Van via the electron transfer pathway. In addition, it was noted that the count was substantially reduced, indicating the agglomeration of nanoparticles.

## 4 Conclusion

In conclusion, Polyethyleneimine mediated the controlled and rapid synthesis of vancomycin-functionalized fluorescent Au-NPs for detecting Hg^2+^ in environmental samples. The size of fluorescent Au-NPs (~7 nm) and the zeta potential value (~38 mV) suggested high fluorescence and stability. The physico-chemical properties of the fluorescent PEI-f-AuNP@Van were determined using X-ray photoelectron spectroscopy, UV–vis spectroscopy, X-ray diffraction, transmission microscopy, and photoluminescence spectroscopy. The synthesized fluorescent Au-NPs demonstrated a strong binding affinity for Hg^2+^, which was associated with a low detection limit of 0.988 nM and the capability for detecting Hg^2+^ in environmental samples with high selectivity and sensitivity. This study indicates possibilities for developing low-cost, ultra-sensitive, and straightforward detection methods for Hg^2+^.

## Data Availability

The original contributions presented in the study are included in the article/Supplementary Material, further inquiries can be directed to the corresponding authors.
